# One-Pot Synthesis of 2′-Aminobenzothiazolo-Arylmethyl-2-Naphthols Catalyzed by NBS under Solvent-Free Conditions

**DOI:** 10.1155/2013/702929

**Published:** 2013-05-30

**Authors:** Wei Lin Li, Li Li Wang, Qiu Yan Luo

**Affiliations:** ^1^Department of Medicinal Chemistry, Pharmacy College of Xinxiang Medical University, Xinxiang 453003, China; ^2^Department of Gynecological and Obstetric, The Third Affiliated Hospital of Xinxiang Medical College, Xinxiang 453003, China

## Abstract

To develop a new facile protocol for the synthesis of 2′-aminobenzothiazolo-arylmethyl-2-naphthol derivatives, N-bromosuccinimide (NBS) was used as an efficient catalyst for the one-pot synthesis of 2′-aminobenzothiazolo-arylmethyl-2-naphthols in excellent yields from **β**-naphthol (1 mmol), aromatic aldehydes (1 mmol), and 2-aminobenzothiazole (1 mmol) at 60°C under solvent-free conditions.

## 1. Introduction

Organic reactions under solvent-free conditions have attracted much interest of chemists particularly from the viewpoint of green chemistry. Green chemistry approaches are significant due to the reduction in byproducts, reduction in produced waste, and reduction of energy cost. The possibility of performing multicomponent reactions under solvent-free conditions with a heterogeneous catalyst could enhance their efficiency from an economic as well as an ecological point of view [1–5]. 

The synthesis of new heterocyclic compounds has always been a subject of great interest due to their wide applicability. Heterocyclic compounds occur very widely in nature and are essential to life. Amongst a large variety of heterocyclic compounds, heterocycles containing benzothiazole moiety are of interest because they show some pharmacological and biological activities. Benzothiazole derivatives were reported to possess anti-inflammatory [6], anti-tumour [7–9], anticonvulsant [10, 11], antibacterial [12], antifungal [13], and topoisomerase II inhibitory activities [14]. Thus, the synthesis of benzothiazole is an important and useful task in organic chemistry. In recent years, the synthesis of 2′-aminobenzothiazolo-arylmethyl-2-naphthols has been reported using LiCl [15] and [Hnmp] HSO_4_ [16] as catalysts.

The use of organic molecules as catalysts has become an attractive alternative to traditional metal catalysts. Interest in the field of organocatalysis has increased spectacularly in the last few years as the result of both the novelty of the concept and, more importantly, the fact that the efficiency and selectivity of many organocatalytic reactions meet the standards of established organic reactions [17]. NBS is one such catalyst, which has recently received considerable attention as a catalyst in various organic transformations [18–25], and is widely used as a brominating reagent. Furthermore, it is also used in oxidation and free radical reactions under mild and convenient conditions to afford the desired products in excellent yields and with high selectivities. However, there are no examples of the use of NBS as a catalyst for the synthesis of 2′-aminobenzothiazolo-arylmethyl-2-naphthols.

**Scheme 1:**
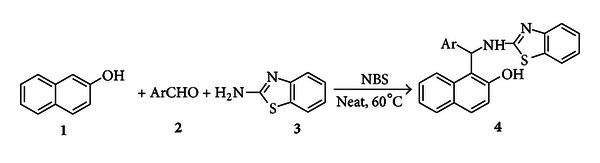


In continuation of our efforts to explore newer reactions for the synthesis of heterocyclic compounds, we wish to report here a facile and improved protocol for preparation of 2′-aminobenzothiazolo-arylmethyl-2-naphthols from *β*-naphthol, aromatic aldehydes, and 2-aminobenzothiazole in the presence of NBS as a catalyst under solvent-free conditions (Scheme 1).

## 2. Results and Discussion

Initially, we decided to explore the role of our catalyst in water and ethanol-water (1 : 1) as solvent system for the synthesis of 2′-aminobenzothiazolo-phenylmethyl-2-naphthol used as a model compound. With respect to the solvent system, the best results were achieved using water (Table 1, Entry 2). In recent years, the synthesis of compounds under solvent-free conditions is an important task in heterocyclic synthesis. Therefore, we decided to test this solvent-free reaction with various ratios of catalysts. We found that the reaction was rapid and gave excellent yields of the products when using 10 mol% NBS.

These results encouraged us to investigate the scope and generality of this new protocol for various aromatic aldehydes under optimized conditions. As shown in Table 2, a series of aromatic aldehydes containing either electron-withdrawing or electron-donating substituents successfully react with *β*-naphthol and 2-aminobenzothiazole which afforded good to high yields of products with high purity, at 60°C under solvent-free conditions.

**Scheme 2:**
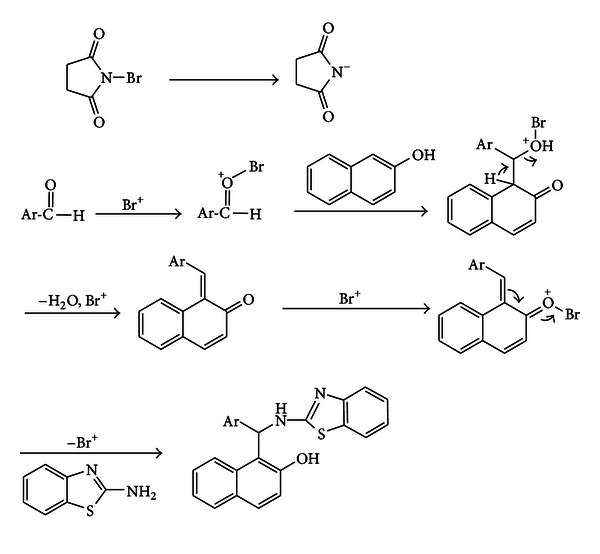


It is likely that the reagent releases Br^+^ *in situ*, which can act as an electrophilic species. Therefore, the mechanism shown in Scheme 2 can be suggested for the conversion of the *β*-naphthol 2-aminobenzothiazole and various aromatic aldehydes to 2′-aminobenzothiazolo-arylmethyl-2-naphthols.

In summary, we have developed a new facile protocol for the synthesis of 2′-aminobenzothiazolo-arylmethyl-2-naphthol derivatives from the reaction of *β*-naphthol, aromatic aldehydes, and 2-aminobenzothiazole compounds using NBS under solvent-free conditions.

## 3. Experimental Part

*General.* IR spectra were determined on FTS-40 infrared spectrometer; NMR spectra were recorded on Bruker AV-400 spectrometer at room temperature using TMS as an internal standard; coupling constants (*J*) were measured in Hz; elemental analysis was performed by a Vario-III elemental analyzer; melting points were determined on an XT-4 binocular microscope and were uncorrected; commercially available reagents were used throughout without further purification unless otherwise is stated.

### 3.1. General Procedure for the Preparation of **4**

A mixture of the *β*-naphthol (1 mmol), aldehydes (1 mmol), 2-aminobenzothiazole (1 mmol), and NBS (0.1 mmol) was stirred at 60°C for the appropriate time according to Table 2. Completion of the reaction was indicated by TLC. The reaction was cooled to room temperature, washed with water, and extracted with ethyl acetate (3 × 10 mL). The combined organic layers were dried over sodium sulfate and concentrated under reduced pressure to afford a white powder. The pure solid products were obtained by recrystallization from ethanol.

### 3.2. Spectral Data of New Products

2′*-Aminobenzothiazolo-(3-nitrophenyl)methyl-2-naphthol *(**4g**). IR (KBr): *v* 3335 (–OH), 1627 (C=N), 1597 (aromatic C=C), 1541, 1531 (aromatic C=C), 1452, 1436, 1347, 1312, 1270, 1252, 1210, 813, 753 cm^−1^; 1H NMR (DMSO-d6, 400 MHz) *δ*: 7.05–7.88 (m, 15H, ArH and CH), 8.90 (s, 1H, NH), 10.12 (brs, 1H, OH, D2O, exchangeable); Anal. calcd. for C24H17N3O3S: C 67.43, H 4.01, N 9.83, S 7.50; found C 67.50, H 4.00, N 9.87, S 7.42.

2′*-Aminobenzothiazolo-(3,4,5-trimethoxyphenyl)methyl-2-naphthol *(**4k**). IR (KBr): *v* 3347 (–OH), 2936, 2836, 2599, 1627 (C=N), 1595 (aromatic C=C), 1544, 1507 (aromatic C=C), 1436, 1417, 1332, 1268, 1233, 1129, 1006, 816, 753 cm^−1^; ^1^H NMR (DMSO-*d*_6_, 400 MHz) **δ**: 3.62 (s, 9H, OCH_3_), 6.80–7.81 (m, 13H, ArH and CH), 8.81 (s, 1H, NH), 10.15 (s, 1H, OH, D_2_O, exchangeable); Anal. calcd. for C_27_H_24_N_2_O_4_S: C 68.62, H 5.12, N 5.93, S 6.79; found C 68.70, H 5.04, N 6.00, S 6.82.

## Figures and Tables

**Table 1:** The synthesis of 2′-aminobenzothiazolo-phenyl-2-naphthol in various conditions.

Entry	Solvent	NBS (mol%)	Temperature (°C)	Time (min)	Yield (%)
1	Water	10	25	90	59
2	Water	10	60	60	88
3	Ethanol-water	10	25	60	56
4	Ethanol-water	10	60	30	85
5	Neat	0	60	60	0
6	Neat	2	60	30	73
7	Neat	5	60	20	89
8	Neat	10	25	60	65
9	Neat	10	60	10	94
10	Neat	15	60	10	92
11	Neat	20	60	10	92

**Table 2:** Preparation of 2′-aminobenzothiazolo-arylmethyl-2-naphthols^a^.

Entry	Ar	Time (min)	Products	Yield (%)^b^	m.p. (lit.) (°C)^c^
1	C_6_H_5_	10	**4a**	94	202–204 (204-205) [15]
2	4-Cl-C_6_H_4_	8	**4b**	95	209-210 (209-210) [15]
3	4-Me-C_6_H_4_	10	**4c**	92	183-184 (182-183) [15]
4	4-F-C_6_H_4_	10	**4d**	98	189-190 (188-189) [16]
5	4-NO_2_-C_6_H_4_	5	**4e**	97	188-189 (190-191) [16]
6	4-MeO-C_6_H_4_	10	**4f**	90	172-173 (175-176) [16]
7	3-NO_2_-C_6_H_4_	8	**4g**	93	197–199 (198-199) [15]
8	2-Cl-C_6_H_4_	8	**4h**	91	185-186 (189-190) [16]
9	2,4-Cl_2_-C_6_H_4_	8	**4i**	96	203-204 (206-207) [16]
10	4-OH-3-MeO-C_6_H_3_	15	**4j**	88	192–194 (194-195) [16]
11	3,4,5-MeO_3_C_6_H_2_	15	**4k**	92	159-160

^a^Reaction conditions: *β*-naphthol (1 mmoL), aldehyde (1 mmoL), 2-amino-benzothiazole (1 mmoL), NBS (0.1 mmoL), 60°C, and neat.

^b^Isolated yield.

^c^Temperature value in the reference.

## References

[1] Kumar A, Maurya RA (2007). Synthesis of polyhydroquinoline derivatives through unsymmetric Hantzsch reaction using organocatalysts. *Tetrahedron*.

[2] Tietze LF, Kinzel T, Brazel CC (2009). The domino multicomponent allylation reaction for the stereoselective synthesis of homoallylic alcohols. *Accounts of Chemical Research*.

[3] Tietze LF, Beifuss U (1993). Sequential transformations in organic chemistry: a synthetic strategy with a future. *Angewandte Chemie—International Edition*.

[4] Bienaymé H, Hulme C, Oddon G, Schmitt P (2000). Maximizing synthetic efficiency: multi-component transformations lead the way. *Chemistry*.

[5] Reddy UC, Bondalapati S, Saikia AK (2009). Stereoselective one-pot, three-component synthesis of 4-aryltetrahydropyran via Prins-Friedel-Crafts reaction. *Journal of Organic Chemistry*.

[6] El-Shorbagi AN, Sakai SI, El-Gendy MA, Omar N, Farag HH (1989). Imidazo[2,1-b]benzothiazoles, II. Synthesis and antiinflammatory activity of some imidazo[2,1-b]benzothiazoles. *Chemical and Pharmaceutical Bulletin*.

[7] Shi DF, Bradshaw TD, Wrigley S (1996). Antitumor benzothiazoles. 3. Synthesis of 2-(4-aminophenyl)benzothiazoles and evaluation of their activities against breast cancer cell lines in vitro and in vivo. *Journal of Medicinal Chemistry*.

[8] Wells G, Bradshaw TD, Diana P (2000). Antitumour benzothiazoles—part 10: the synthesis and antitumour activity of benzothiazole substituted quinol derivatives. *Bioorganic and Medicinal Chemistry Letters*.

[9] Hutchinson I, Chua MS, Browne HL (2001). Antitumor benzothiazoles. 14.^1^ synthesis and in vitro biological properties of fluorinated 2-(4-aminophenyl)benzothiazoles. *Journal of Medical Chemistry*.

[10] Chopade RS, Bahekar RH, Khedekar PB, Bhusari KP, Rao AR (2002). Synthesis and anticonvulsant activity of 3-(6-substituted-benzothiazol-2-yl)-6-phenyl-[1, 3]-xazinane-2-thiones. *Archiv Der Pharmazie*.

[11] Amnerkar ND, Bhusari KP (2010). Synthesis, anticonvulsant activity and 3D-QSAR study of some prop-2-eneamido and 1-acetyl-pyrazolin derivatives of aminobenzothiazole. *European Journal of Medicinal Chemistry*.

[12] Palkar M, Noolvi M, Sankangoud R, Maddi V, Gadad A, Nargund LVG (2010). Synthesis and antibacterial activity of a novel series of 2,3-diaryl-substituted-imidazo(2,1-b)-benzothiazole derivatives. *Archiv der Pharmazie*.

[13] Singh T, Srivastava VK, Saxena KK, Goel SL, Kumar A (2006). Synthesis of new thiazolylthiazolidinylbenzothiazoles and thiazolylazetidinylbenzothiazoles as potential insecticidal, antifungal, and antibacterial agents. *Archiv der Pharmazie*.

[14] Choi SJ, Park HJ, Lee SK, Kim SW, Han G, Choo HYP (2006). Solid phase combinatorial synthesis of benzothiazoles and evaluation of topoisomerase II inhibitory activity. *Bioorganic and Medicinal Chemistry*.

[15] Shaabani A, Rahmati A, Farhangi E (2007). Water promoted one-pot synthesis of 2′-aminobenzothiazolomethyl naphthols and 5-(2′-aminobenzothiazolomethyl)-6-hydroxyquinolines. *Tetrahedron Letters*.

[16] Yu Y, Guo H (2011). One-pot synthesis of 2′-aminobenzothiazolo-arylmethyl-2-naphthols in ionic liquid of [Hnmp]HSO4under solvent-free conditions. *Chinese Journal of Organic Chemistry*.

[17] Dalko PI, Moisan L (2004). In the golden age of organocatalysis. *Angewandte Chemie—International Edition*.

[18] Karimi B, Seradj H, Ebrahimian GR (2000). Mild and efficient conversion of aldehydes to 1,1-diacetates catalyzed with N-bromosuccinimide. *Synlett*.

[19] Hazarkhani H, Karimi B (2004). N-bromosuccinimide as an almost neutral catalyst for efficient synthesis of dihydropyrimidinones under microwave irradiation. *Synthesis*.

[20] Kuo CW, More SV, Yao CF (2006). NBS as an efficient catalyst for the synthesis of 1,5-benzodiazepine derivatives under mild conditions. *Tetrahedron Letters*.

[21] Hajipour AR, Pourmousavi SA, Ruoho AE (2006). Chemoselective and solvent-free thioacetalization of aldehydes by a catalytic amount of NBS. *Synthetic Communications*.

[22] Khazaei A, Rostami A, Raiatzadeh A, Mahboubifar M (2007). N-bromosuccinimide (NBS)-selective and effective catalyst for trimethylsilylation of alcohols and phenols using hexamethyldisilazane and their regeneration under mild and neutral reaction conditions. *Canadian Journal of Chemistry*.

[23] Khazaei A, Rostami A, Raiatzadeh A (2007). N-bromosuccinimide (NBS): a mild and efficient catalyst for tetrahydropyranylation of alcohols and phenols under solvent-free conditions. *Journal of the Chinese Chemical Society*.

[24] Rostami A, Jafari H (2008). NBS as a powerful catalyst for the synthesis of *β*-hydroxysulphides with thiolysis of epoxides under mild reaction conditions. *South African Journal of Chemistry-suid-afrikaanse Tydskrif vir Chemie*.

[25] Shaterian HR, Yarahmadi H, Ghashang M, Safari MM (2008). N-bromosuccinimide catalyzed one-pot and rapid synthesis of acetamidobenzyl naphthols under mild and solvent-free conditions. *Chinese Journal of Chemistry*.

